# Early initiation of antiretroviral therapy results in decreased morbidity and mortality among patients with TB and HIV

**DOI:** 10.1186/1758-2652-12-14

**Published:** 2009-07-16

**Authors:** Payam Tabarsi, Ali S Saber-Tehrani, Parvaneh Baghaei, Mojgan Padyab, Davood Mansouri, Majid Amiri, Mohammad Reza Masjedi, Frederick L Altice

**Affiliations:** 1 Mycobacteriology Research Center, National Research Institute of Tuberculosis and Lung Disease, Shahid Beheshti University of Medical Science, Tehran, Iran; 2 Yale University AIDS Program, New Haven, CT, USA

## Abstract

**Introduction:**

The overlapping drug toxicity profiles, drug-drug interactions and complications of management of both HIV and tuberculosis (TB) in patients with advanced HIV have not been fully delineated.

**Methods:**

We conducted a retrospective chart review of the outcomes of tuberculosis treatment among 69 HIV-infected patients with TB, who were hospitalized in Masih Daneshvari Hospital in Tehran, Iran between 2002 and 2006, and who received standard category 1 (CAT-1) regimens. Group I (N = 47) included those treated from 2002 to 2005 with highly active antiretroviral therapy (HAART) initiated after eight weeks of TB treatment for those whose CD4 count was <200 cells/mm^3^. Group II (N = 22) included TB patients treated from 2005 to 2006, with HAART initiated after two weeks of TB treatment if their CD4 count was <100 cells/mm^3 ^and eight weeks after initiation of TB treatment for those whose CD4 count was between 101 and 200 cells/mm^3^.

**Results:**

There were no differences between Groups I and II with regard to: adverse drug reactions [four (8.5%) versus two (9%), p = ns]; IRIS [six (12.7%) versus three (10.7%), p = ns]; and new opportunistic infections [eight (17.0%) versus two (9.1%), p = ns]. Death, however, occurred more frequently in Group I than in Group II [13 (27.7%) versus (4.5%), p = 0.03], where HAART was initiated earlier. Injection of drugs was the most common route of HIV transmission in both groups (72.3% in Group I and 77.3% in Group II).

**Conclusion:**

This manuscript shows that in a retrospective review of HIV/TB patients hospitalized in Tehran, improved survival was associated with earlier initiation of antiretroviral therapy in HIV/TB patients with CD4 counts of below 100 cells/mm^3^.

## Findings

Multiple complications in the co-management of HIV and TB have been documented or suggested, including: overlapping medication toxicity profiles; pharmacokinetic drug interactions; premature death from non-tuberculosis causes; development of resistant TB; and immune reconstitution reactions [1-3]. Recent data suggest that, with careful attention to these complicating factors, the mortality associated with HIV-related TB is substantially reduced with the provision of HAART [4], yet data conflict with regard to CD4 count cut-offs and consequences of the immune reconstitution syndrome [5].

In this study, we compare the clinical treatment outcomes of HIV and TB co-infected patients treated with HAART, considering a delayed versus immediate HAART approach among hospitalized patients in an academic medical centre in Tehran, Iran.

We conducted a retrospective chart review of all HIV-positive patients diagnosed with tuberculosis from 2002 to 2006 at the Masih Daneshvari Medical Center; the chart review continued for an additional year to evaluate clinical outcomes.

From 2002 to 2005, the Iranian national guidelines for management of TB among HIV-positive patients included initiation of HAART eight weeks after initiation of treatment for TB if patients' CD4 count was <200 cells/mm^3^. In 2005, our centre changed its guidelines to initiate HAART concurrently with TB treatment if the CD4 count was <100 cells/mm^3 ^and to delay treatment of HIV for eight weeks if the CD4 count was 101 to 200 cells/mm^3^. During this time period, all TB patients received anti-tuberculosis medications as directly observed therapy and in accordance with standard Category 1 (CAT-1) regimens.

Group I was made up of patients receiving TB treatment according to the time period stipulated by the previous guidelines; Group II consisted of those treated in accordance with the newer guidelines. Based on the Drug Susceptibility Test (DST), the regimen was changed according to drug resistance pattern.

Tuberculosis treatment outcomes were measured in accordance with standard WHO definitions [6]. HIV treatment strategies differed during the two treatment periods as well. Treatment outcomes were defined as those identified within 12 months after diagnosis of TB. The presence of a new opportunistic infection diagnosed during the first 12 months after diagnosis included only those opportunistic infections that were new for the patient.

Medication adverse consequences were documented from the medical record, and laboratory grading of adverse events were in accordance with the ACTG guidelines [7]. Immune reconstitution syndrome was defined according to criteria proposed by French et al [8].

From 2002 to 2005, HAART was initiated with zidovudine, lamivudine and nelfinavir. At the time of HAART initiation, administration of rifampin, as part of CAT-1 treatment, was changed to rifabutin, 150 mg daily. From 2005 to 2006, efavirenz became available and was dosed at 600 mg per day, and rifampin was continued.

Prophylactic regimens against opportunistic infections were provided in accordance with national guidelines during both time periods of the study. This included trimethoprim/sulfamethoxazole for CD4 counts of <200 cells/mm^3 ^and azithromycin, 1200 mg weekly, for CD4 counts of <50 cells/mm^3^.

For the purposes of this study, we developed a definition of adverse consequence as the development of a Grade 3 or greater laboratory event, development of a new opportunistic infection or immune reconstitution syndrome (IRS), or death. The research was approved and conducted in accordance with an institutional review board.

Statistical data analysis was performed using SPSS v. 13.0 software (Apache Software Foundation, Chicago, Illinois). Chi square testing was performed to determine the bivariate differences between comparisons. The Student's *t*-test and Mann-Whitney U test were used for variables with normal distribution and non-normal distribution data respectively. Logistic regression was used to adjust baseline demographic characteristics between the two groups. Kaplan-Meier survival curves were created for time to death.

The baseline demographic and clinical characteristics of subjects in Groups I and II are provided in Table 1. The mean age in Group I and Group II was 33.3 ± 7.9 and 37.8 ± 6.3 respectively. All of patients in Group I were male.

**Table 1:** Baseline characteristics of subjects

Subject characteristics	Group I 2002–2005	%	Group II 2005–2007	%	P value
	N = 47		N = 22		
Mean age (years)	33.3 ± 7.9		37.8 ± 6.3		0.016
Gender					
• Male	44	93.6	22	100	
• Female	3	6.4	0	0	
HIV diagnosis at hospital	43	91.5	16	72.7	0.064
Prior OI (%)	0	0	0	0	
HBV co-infected	2	4.3	2	9.1	0.587
HCV co-infected	38	80.9	15	68.2	0.245
Type of TB					
• Pulmonary only	31	66	19	86.4	0.08
• Disseminated	16	34	3	13.6	

TB drug susceptibility					

• Unknown	18	38.3	7	31.8	0.485

• Pan-sensitive	15	31.9	11	50	

• Single drug resistance	7	14.9	3	13.6	

• Poly-drug resistance	4	8.5	0	0	

• Multi-drug resistance	3	6.4	1	4.5	

Mean CD4 lymphocyte count at diagnosis (+/- SD)	204 ± 184.06		164.7 ± 200.9		0.20

• Median CD4 lymphocyte count	132		60		

• CD4 ≤ 100 cells/mm^3^	15		13		

• CD4 101–200 cells/mm^3^	26		9		0.22

Injection drug use was the most common route (73.9%) of HIV transmission, and most of the patients (87%) had a history of imprisonment. The mean CD4 count was 204 ± 184.06 in Group I. The median CD4 count statistically differed between the two groups, as did the proportion with extra-pulmonary TB. Baseline resistance did not differ between the two groups. The number of patients co-infected with the hepatitis C virus (HCV) was 38 and 15 in Group I and Group II respectively.

**Figure 1:**
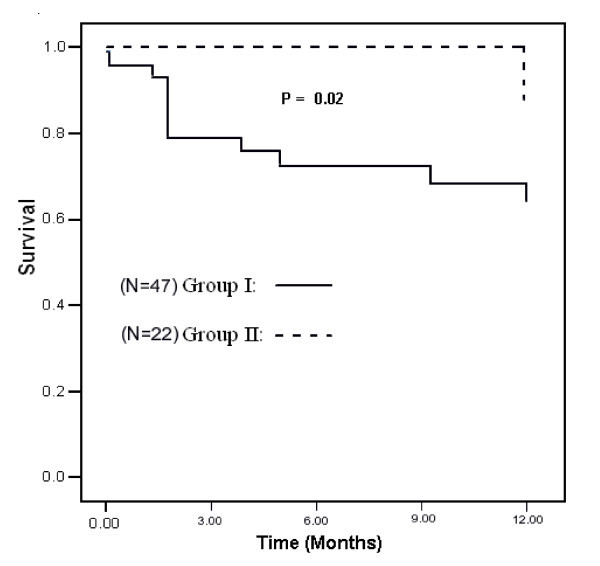
**Kaplan-Meier curve of HIV/TB co-infected patients and time to death**.

**Figure 2:**
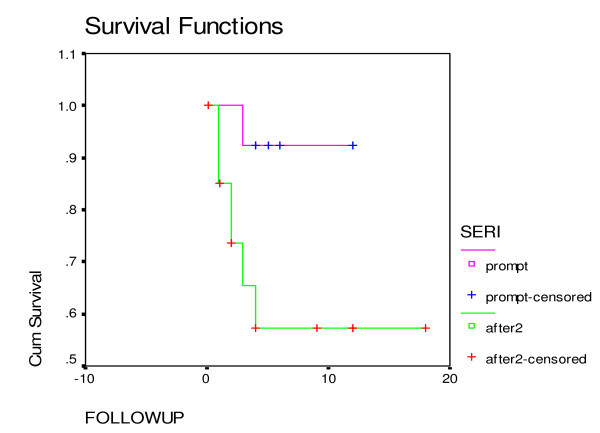
**Kaplan-Meier curve of HIV/TB co-infected patients with CD4 count less than 100 cells/mm^3 ^(p value = 0.0335)**.

Table 2 provides the treatment outcomes of the two groups. The most common adverse reaction was drug-induced hepatitis, which occurred in 14.5% of the patients. The proportion of subjects with IRS was 13%, and adverse cutaneous reactions occurred in 7.7% of subjects. No difference was observed between the two groups for any of these complications of therapy. The Kaplan-Meier curve for HIV and TB co-infected patients is shown in Figures 1 and 2. Death, within 12 months after diagnosis, was 7.2 times more likely in Group I than in Group II (95% confidence interval 1.87–27.64, p value = 0.03).

**Table 2:** Clinical responses to treatment

Response to treatment	Group I (N = 47) 2002–2005		Group II (N = 22) 2005–2007		P value
	N	%	N	%	

Initial HAART regimen					

• Nelfinavir-containing regimen	47	100			

• Efavirenz-containing regimen			22	100	

Discontinued HAART within one year of initiation					

Laboratory ASEs* (Grade 3 or 4)					

• Grade 3	4	8.5	1	4.5	0.9

• Grade 4	0	0	1	4.5	0.319

New opportunistic infections (overall)	8	17.0	2	9.1	0.484

• CNS toxoplasmosis	6	12.8	0	0	

• CMV retinitis	1	2.1	0	0	

• PCP pneumonia	0	0	2	9.1	

• Cryptococcal infection	1	2.1	0	0	

TB outcomes					

• Cure	22	46.8	19	86.4	0.002

• Treatment failure	12	25.5	2	9.1	0.198

Death within 12 months	13	27.7	1	4.5	0.028

• Median time to death (days)	60		NA		

* Adverse side effects

The causes of death in Group 1 were TB (10 cases), and primary CNS lymphoma, CMV and cryptococal meningitis (one case for each). TB was the cause of the only death in Group II. The CD4 count median among patients who died in Group I was 77.5 cells/mm^3^, while the only patient who died in Group II had a CD4 count of 52 cells/mm^3^. The outcome for patients, based on the DST pattern, is shown in Table 3.

**Table 3:** Outcomes of patients based on DST pattern

DST	Outcome of Group 1 (N = 47)			Outcome of Group 2 (N = 22)		
	
	Cure	Death	Failure	Cure	Death	Failure
Unknown	5 (22.7%)	8 (61.5%)	5 (41.7%)	5 (26.3%)	1 (100%)	1 (50%)
Sensitive	10 (45.5%)	3 (23.1%)	2 (16.7%)	11 (57.9%)	0	0
Mono-drug resistant	4 (18.2%)	1 (7.7%)	2 (16.7%)	2 (10.5%)	0	1 (50%)
Poly-drug resistant	2 (9.1%)	0	2 (16.7%)	0	0	0
MDR	1 (4.5%)	1 (7.7%)	1 (8.3%)	1 (5.3%)	0	0

Though the study size is small and limited by non-contemporaneous treatment approaches, this study suggests that immediate treatment with antiretroviral therapy in patients with TB and advanced HIV markedly improves survival without increasing adverse consequences (Figure 2). However, the study could not confirm this because: the two groups received different treatment at different times; and many other factors may have influenced the result, such as changing antiretroviral therapy from nelfinavir to efavirenz. The definitive answer to the optimal timing of ART requires a randomized clinical study.

Nevertheless, the study's suggestion is particularly relevant in that nearly three quarters of this study population acquired HIV from injection drug use, and were highly likely to be co-infected with HCV. In other studies of treatment for HIV among HCV co-infected patients, development of Grade 3/4 hepatotoxicity is markedly increased. This study, however, suggests little, if any, excess hepatotoxicity compared to other similar study populations. In other studies of treatment for HIV and TB, the presence of Grade 3/4 hepatotoxicity ranges from 6% to 18% in non-drug-using populations [9].

Our study, with nearly three quarters acquiring HIV infection from injection drug use, suggests the safety of TB and HIV treatment among injection drug users. Though IRS has been reported in as many as 30% to 40% of TB patients starting HAART, this study, in line with some recent publications [10,11], suggests that this occurs less frequently than previously reported and does not result in discontinuation of either HIV or TB treatment. The proportion of patients with rash in our study is similar to that reported among patients treated with non-nucleoside reverse transcriptase inhibitors (NNRTIs) who were not TB co-infected [12]. In our study, no one discontinued treatment for either IRS or rash; however, a few patients' NNRTIs were changed to alternative regimens.

While consensus is beginning to develop, the appropriate time to initiate HAART in TB/HIV patients remains controversial [13], and it will remain so until systematic and prospective randomized controlled trials can be completed. While other studies demonstrate the markedly increased risk for death among TB/HIV patients whose HIV is not treated when the CD4 count is ≤ 50 cells/mm^3^, our study suggests that benefit is derived with earlier treatment for CD4 counts that are up to 100 cells/mm^3^.

Though the sample size was small and did not achieve statistical significance, this study confirms the high rate of opportunistic infections among those initiating HAART after eight weeks of TB treatment.

Surprising in our data was the observation of high rates of toxoplasmosis and pneumocystis pneumonia (PCP) among a group on TMP/SMZ for PCP prophylaxis [14]. One explanation is that patients who were prescribed PCP prophylaxis did not take it. While others have noted that non-adherence to one medication is associated with non-adherence to another [15], patients received their TB medications as directly observed therapy while their prophylaxis and HIV medications were self-administered.

Though not a primary goal of this retrospective study, the high rate of HIV diagnosis coincident with TB diagnosis among injection drug users and former prisoners provides a strong argument for more routine testing of these individuals. Prior identification of HIV may have led to: increased INH preventive therapy; earlier initiation of HAART and possibly avoiding development of TB and other opportunistic infections; engagement in methadone treatment for stabilization of opioid dependence; engagement in health; and less exposure to TB in congregate settings like prisons [16,17].

Although this study was retrospective and had limitations, such as being hospital based and having a limited number of patients, overall it marks the importance of rapid initiation of HAART in TB/HIV patients with advanced HIV infection (CD4 count of <100 cell/mm^3^).

## Competing interests

The authors declare that they have no competing interests.

## Authors' contributions

PT participated in the design of the study, collecting data, analyzing and preparing the manuscript. AS participated in collecting data and preparing the manuscript. PB participated in collecting data and analyzing data. MP participated in analyzing data. MA participated in the design of the study. DM participated in the design of the study and preparing the manuscript. MM participated in the design of the study. RA participated in the design of the study and preparing the manuscript. All authors read and approved the final manuscript.
